# Comparative Phytochemical Analysis of Essential Oils from Different Biological Parts of *Artemisia herba alba* and Their Cytotoxic Effect on Cancer Cells

**DOI:** 10.1371/journal.pone.0131799

**Published:** 2015-07-21

**Authors:** Mounir Tilaoui, Hassan Ait Mouse, Abdeslam Jaafari, Abdelmajid Zyad

**Affiliations:** Laboratory of Biological Engineering, Natural Substances, Cellular and Molecular Immuno-pharmacology, Immunobiology of Cancer Cells Cluster, Faculty of Science and Technology, Sultan Moulay Slimane University, Beni-Mellal, Morocco; University of Colorado Denver, UNITED STATES

## Abstract

**Purpose:**

Carrying out the chemical composition and antiproliferative effects against cancer cells from different biological parts of *Artemisia herba alba*.

**Methods:**

Essential oils were studied by gas chromatography coupled to mass spectrometry (GC–MS) and their antitumoral activity was tested against P815 mastocytoma and BSR kidney carcinoma cell lines; also, in order to evaluate the effect on normal human cells, oils were tested against peripheral blood mononuclear cells PBMCs.

**Results:**

Essential oils from leaves and aerial parts (mixture of capitulum and leaves) were mainly composed by oxygenated sesquiterpenes 39.89% and 46.15% respectively; capitulum oil contained essentially monoterpenes (22.86%) and monocyclic monoterpenes (21.48%); esters constituted the major fraction (62.8%) of stem oil. Essential oils of different biological parts studied demonstrated a differential antiproliferative activity against P815 and BSR cancer cells; P815 cells are the most sensitive to the cytotoxic effect. Leaves and capitulum essential oils are more active than aerial parts. Interestingly, no cytotoxic effect of these essential oils was observed on peripheral blood mononuclear cells.

**Conclusion:**

Our results showed that the chemical composition variability of essential oils depends on the nature of botanical parts of *Artemisia herba alba*. Furthermore, we have demonstrated that the differential cytotoxic effect depends not only on the essential oils concentration, but also on the target cells and the botanical parts of essential oils used.

## Introduction

*Artemisia herba-alba* (Asteraceae) is a greenish-silver perennial dwarf shrub growing in arid and semi-arid climates. It occurs through the Mediterranean region in North Africa, Spain, deserts of Sinai Peninsula, the Middle East, Northwestern Himalayas, and in India. *Artemisia herba alba* Asso (desert wormwood, armoise blanche (Fr.), chich (Arab.) is a dwarf shrub with a rapid growth in dry and warm climates and in muddy areas [1].The various species are morphologically different from each other depending on geographical, environmental, and climatic conditions. The plant is green to light green, with strong, sturdy roots. The flowering time and harvesting is around May/June and continues until October in some areas [2]. This plant is used as a flavouring and as a medicine herb for the treatment of colds, coughing, intestinal disturbances and as an antidiabetic agent [3, 4]. Investigations on the medicinal properties of *Artemisia herba alba* extracts reported anti-diabetic, leishmanicidal, antibacterial, and antifungal properties [5, 6]. Over the last decades, studies on *Artemisia herba alba* were focused on its essential oils. Their composition through the world revealed a high level of polymorphism and led to the definition of several chemotypes. Because of their importance in the fragrance industry, numerous studies on *Artemisia herba alba* essential oils have been published [7, 8]. Two reviews are also available [9, 10].

In Spain, essential oil from *Artemisia herba alba* [8, 11] showed that monoterpene hydrocarbons and oxygenated monoterpenes are the most abundant skeletons, but large amounts of sesquiterpenes also were found in some populations. Camphor, 1,8-cineole, *p*-cymene and davanone were the major chemical compounds found. Two oil types were found for plants grown in Israel and Sinai those of cineole-thujane bornane type and the pinane type with monoterpene skeletons [8].

In Jordan, regular monoterpenes were predominant and the principal components were α- and β-thujones, classifying the *Artemisia herba alba* as being a thujone chemotype [12]. In Morocco, sixteen chemotypes were found, and twelve of them have monoterpenes as major components of essential oils. The remaining four chemotypes, have sesquiterpene skeletons as the major fraction. Investigations reported no correlation between chemotypes and geographic distribution [13].

In Algerian essential oil, monoterpenes were the major components, essentially camphor, α- and β-thujones, 1,8-cineole and chrysanthenyl derivatives [2, 14]. In Tunisian oil, oxygenated monoterpenes were found to be the major components of *Artemisia herba alba* oil extracted from aerial parts [15, 16].

This paper reports the chemical composition and the biological activity against cancer cell lines of the essential oils, obtained from different biological parts of *Artemisia herba alba* collected at Er-Rachidiya (Central- East region of Morocco).

## Materials and Methods

### Ethics statement

*Artemisia herba alba* Asso plant was harvested in Imilchil, Errachidia district central Eastern of Morocco (W 5°39′25.43″ - N 32°12′15.059″) in June 2007 (No specific permits were required for the described field studies or for the collection of plant material).The field studies did not involve endangered or protected species.

Human peripheral blood mononuclear cells (PBMCs): the blood samples were collected from the authors of this manuscript (Mounir Tilaoui,Hassan Ait Mouse; Abdeslam Jaafari and Abdelmajid Zyad) under medical surveillance. Approved by Sultan Moulay Slimane University committee, accreditation No 2008/01-2014. The authors receive written informed consent from the blood donors.

### Plant material

*Artemisia herba alba* Asso plant was harvested in Imilchil, Errachidia district, central Eastern region of Morocco (W 5°39′ 25.43″ - N 32°12′ 15.059″) in June 2007 (No specific permits were required for the described field studies or for the collection of plant material). Whole aerial parts (mixture of capitulum and leaves) on isolated leaves, capitulum (flower head) or stems were shade-dried at room temperature with ventilation. The obtained dry matter was isolated separately from the plant, minced and immediately hydrodistilled to obtain essential oils.

### Gas chromatography and mass spectroscopic analysis (GC-MS)

Analytical gas chromatography was carried out using a Trace GC ULTRA gas chromatography system fitted with a VB-5 (Methylpolysiloxane with 5% of phenyl) column (30 m x 0.25 mm, 0.25 μm film thickness). Carrier gas was helium at a flow rate of 1.4 mL/min. Column temperature was initially kept at 40°C for 2min, and then gradually increased to 300°C at a rate of 20°C/min. Samples (1μL, appropriately diluted in ethyl acetate) were injected at 220°C.

Mass spectrometry analysis was carried on a *Polaris Q MS* coupled to GC ULTRA with an ionic trap mass detector operating in the EI mode (70eV). Components identification was done by GC and GC-MS (according to fragmentation patterns) and by using NIST (National Institute of Standards and Technology) MS Search database.

### Cell culture

The murine mastocytoma cell line (P815, ATCC: TIB64) and the kidney carcinoma cell lines of hamsters (BSR, ATCC: CCL10) were kindly donated by the laboratory of Dr. Michel Lepoivre, 841 Institute of Biochemistry, University of Paris XI, France. The cell lines were cultured in DMEM (Dulbecco’s modified Eagle’s medium) supplemented with 10% Heat-inactivated fetal calf serum (Gibco BRL, Cergy Pontoise, France), penicillin G- streptomycin (1%), and 0.2% sodium bicarbonate (Sigma) at 37°C in a humidified atmosphere containing 5% CO_2_.

### Cytotoxicity assay

The starting inoculums of 2 x 10^5^ P815 cells/mL and 4.8 x 10^4^ BSR cells/mL were used in the exponential phase of growth. Cellular viability was determined by the MTT reduction assay using (3- [4,5-dimethylthiazol-2-yl]-2,5-diphenyl tetrazolium bromide, MTT) [17]. BSR and P815 cell lines were harvested from starting cultures at the exponential growth phase. After a phosphate buffer saline (PBS) wash, the harvested cells were poured in flat-bottomed 96-well microtiter plates containing 100 μL of complete medium per well. Then, the cells were treated with essential oils dissolved in Dimethyl sulfoxide (DMSO) with the appropriate concentrations (all essential oils studied here which are dissolved in DMSO, are soluble in culturing media). Control cells were treated with DMSO, at a final concentration 0, 5% (v/v). After 48-h incubation in a humidified atmosphere at 37°C, 5% CO_2_, 20 μL MTT solution (5 mg/mL PBS) was added. After 4-h incubation under the same conditions, the cleavage of MTT to formazan by metabolically active cells was quantified by scanning the plates at 540 and 630 nm using a MultisKan EX apparatus. The cell viability was calculated by the formula:

%viable cells=(OD of essential oils/OD of controlled cells)×100

Where, OD: Optical Density, Controlled cells: DMSO + cells, OD of essential oils: treated cells.

### Effect on human peripheral blood mononuclear cells (PBMCs)

This test was realised in order to evaluate the effect of the essential oils on human normal cells using the MTT colorimetric assay described above. The viability of PBMCs cells was evaluated by trypan bleu dye exclusion using Malassez Haemocytometer. Cell concentrations were determined as the cells were mixed with 0.2% trypan blue and counted microscopically with a Malasez haemocytometer using the following procedure: 10 μL of cell suspension mixed with 40 μl 0.2% trypan blue and 10 μL of the same mixture was transferred to Malassez haemocytometer. The percentage of cell viability was calculated via the formula: % cell viability = viable cells (unstained) / total number of cells x 100. Cell viability before the cytotoxicity experiment was above 98%.

To isolate the PBMCs, blood samples were collected from healthy donors in heparinized tubes. The blood sample was diluted with the same volume of PBS. After that, the diluted blood sample was carefully layered on Ficoll-hypaque (Amersham Pharmacia Biotech AB). The mixture was centrifuged under at 2000tr/mn for 30 mn. The undisturbed lymphocyte layer was carefully transferred out. The lymphocytes were washed twice with phosphate-buffered solution (PBS) and resuspended in DMEM media. Essential oils were added at the same concentrations able to induce a cytotoxic activity against tumor cells (P815 and BSR). The PBMCs were seeded at a density of (1 × 10^5^ cells/mL) in 96 well plates.

### Statistical analysis

All the experiments were performed in three replicate. Data are reported as the mean ± S.D. n = 3. The significance of differences was estimated using Student’s paired t-test. The difference was considered statistically significant when the P-value was less than 0.05. All statistical analyses were performed using STATISTICA software.

## Results and Discussion

### Chemical composition of the essential oil

**Fig 1:**
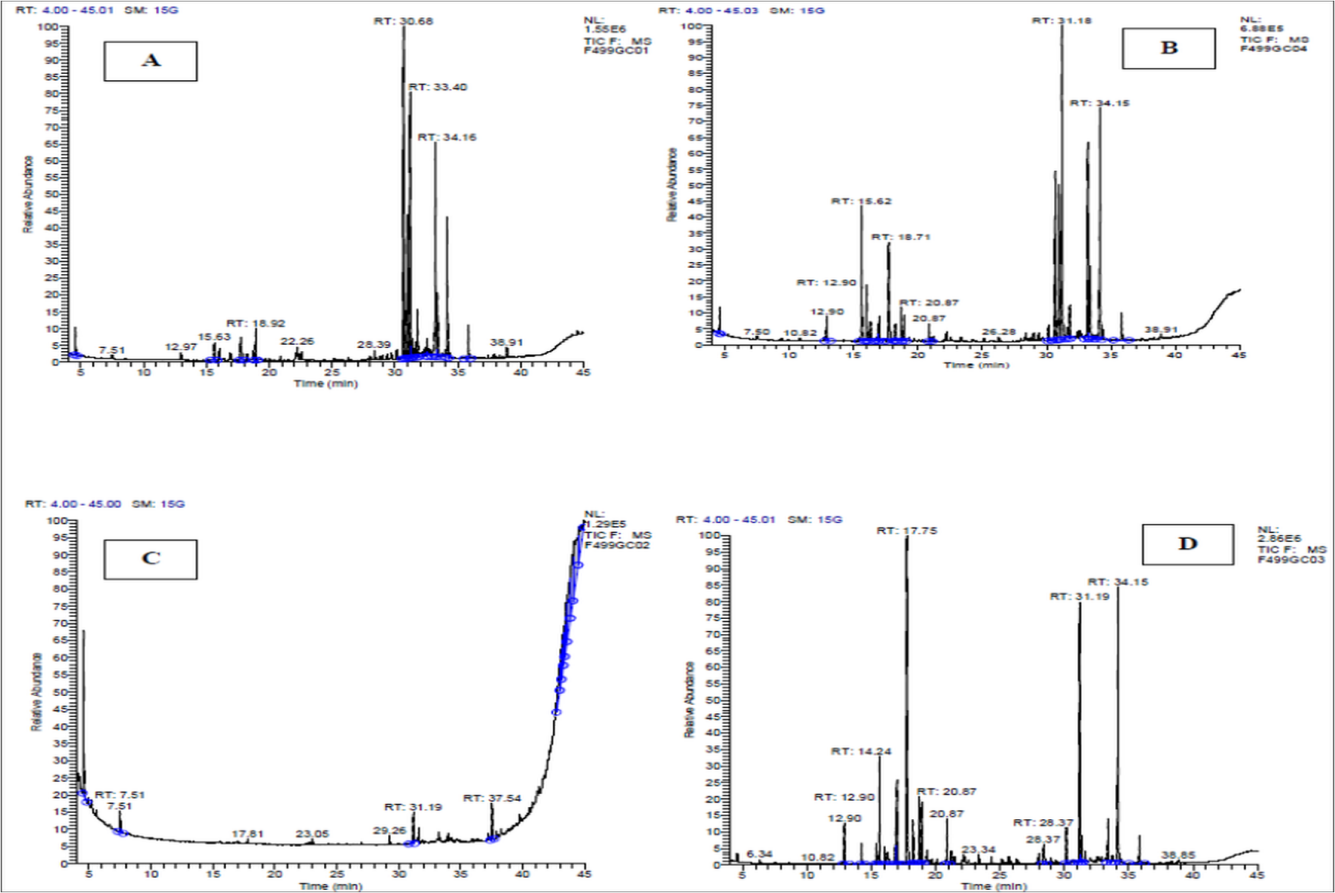
Essential oils chromatogram of different biological parts from *Artemisia herba alba*. A: Leaves, B: Capitulum, C: Stems, D: Aerial parts (Mixture of leaves and capitulum).

The chemical composition of the essential oil was investigated using both GC and GC-MS techniques (Fig 1). Table 1 lists the components identified in the essential oil of different parts of *Artemisia herba alba* (capitulum, leaves, aerial parts and stems). Table 2 shows that essential oil of leaves is composed mainly by oxygenated sesquiterpenes (39.89%), sesquiterpenes (29.04%) and esters (23.97%). Monoterpenes with thujone skeleton (1.24%) and monoterpenes with pinane skeleton (4.48%) are represented by low contents. These compounds are represented by variable concentrations in other parts of the plant studied: β- thujone (1.24%) in leaves and (6.14%) in aerial part, bisabolon oxide (17.55) in aerial parts and 10.27% in leaves. Nevertheless, (+) 2,3,6,7-tetramethyl-, 4,4aα,5,8,8aβ,9β,9aα,10,10aα-decahydroanthracen-9-ol and β-Guaiene are absent in other parts of the plant. In stems essential oil, esters constitute the major fraction with 62.8%. On the other side, essential oil of capitulum is relatively rich in terpenoids compared to other biological parts of the plant; oxygenated monoterpenes and monocyclic monoterpenes are the most abundant with 22.86% and 21.48% respectively. Other important compounds are represented by the sesquiterpenes (12.92%), the monoterpenes with thujane skeleton (10.91%), and the monoterpenes with skeleton pinane (9.24%), while monoterpenes with bornane skeleton were found with low percentage (3.31%).

**Table 1:** Essential oils chemical composition (%) of leaves, stems, capitulum and aerial parts (mixture of capitulum and leaves) of *Artemisia herba alba*.

	Leaves	Stems	Capitulum	Aerial parts	Formula	MW
Monocyclic monoterpenes						
Terpinen-4-ol	-	-	0.89	2.43	C_10_H_16_O	152.23
Piperitone	-	-	1.01	2.69	C_10_H_16_O	152.23
3,5-Heptadienal, 2-ethylidene-6-methyl-	-	-	-	1.74	C_10_H_14_O	150.21
(+-)-2,3,6,7-tetramethyl-1,4,4aα,5,8,8aβ,9β,9aα,10,10aα-decahydroanthracen-9-ol	-	-	19.58	-		
Monoterpenes with thujane skeleton						
β-thujone	1.24	-	7.00	6.14	C_10_H_16_O	152.23
α- thujone	-	-	2.91	-	C_10_H_16_O	152.23
3-thujanol	-	-	1.07	-	C_10_H_16_O	152.23
Cis-sabinol	-	-	-	2.40	C_10_H_16_O	152.23
Thujone	-	-	-	1.01	C_10_H_16_O	152.23
Trans-sabinene hydrate	-	-	-	1.24	C_10_H_20_O	172.26
Monoterpenes with bornane skelton						
Camphor	-	-	1.45	5.12	C_10_H_16_O	152.23
Fenchol	-	-	1.86	3.86	C_10_H_18_O	154.25
Monoterpenes with pinane skelton						
Verbenol	2.16	-	5.99	21.83	C_10_H_16_O	152.24
Myrtenol	2.32	-	1.74	4.19	C_10_H_16_O	152.23
Chrysanthenone	-	-	1.51	-	C_10_H_14_O	150.22
Sesquiterpenes						
Widdrene	9.95	-	-	-	C_15_H_24_	204.35
α-bergamotene	3.31	-	-	-	C_15_H_24_	204.35
α-longipinene	-	-	1.84	-	C_15_H_24_	204.35
β-Guaiene	-	-	11.08	-	C_15_H_24_	204.35
α –bulnesene	15.78	-	-	-	C_15_H_24_	204.35
Ester						
Acetic acid, butyl ester	1.49	10.50	1.19	-	C_6_H_12_O_2_	116.15
2,5-Octadecadiynoic acid, methyl ester	22.48	3.08	9.51	-	C_21_H_38_O_2_	322.52
1-Butanol, 3-methyl-, acetate	-	2.10	-	-	C_7_H_14_O_2_	130.18
[1,1'-Bicyclopropyl]-2-octanoic acid, 2'-hexyl-, methyl ester	-	5.79	-	-	C_21_H_38_O_2_	322.52
Cyclopropaneoctanoic acid, 2-[[2-[(2thylcyclopropyl)methyl]cyclopropyl]methyl]-, methyl ester	-	4.65	-	-	C_22_H_38_O_2_	334.53
Ethyl linoleate	-	19.28	-	-	C_20_H_36_O_2_	308.50
Linoleic acid	-	6.31	-	-	C_18_H_32_O_2_	280.44
13,16-Octadecadiynoic acid, methyl ester	-	11.09	-	-	C_21_H_38_O_2_	322.52
Oxygenated sesquiterpenoids						
Caryophyllene oxide	1.49	-	-	1.76	C_15_H_24_O	220.35
Farnesene epoxide, E	4.97	-	4.38	17.08	C_15_H_24_O	220.35
Bisabolone oxide	10.27	-	13.64	17.55	C_15_H_24_O_2_	236.35
trans-(Z)-α-bisabolene epoxide	-	2.86	-	-	C_15_H_24_O	220.35
1,2–15,16-Diepoxyhexadecane	-		-	-	C_20_H_40_O_2_	312.53
Eucalyptol (1,8-Cineole)	20.37	7.71	1.49	2.27	C_10_H_18_O	154.24
Α-bisabolol oxide A	-	-	2.20	2.26	C_15_H_26_O_2_	238.36
Bergamotol, Z-α-trans	2.79	-	0.97	2.24	C_15_H_24_O	220.35
α-Bisabolol oxide	-	-	-	2.99	C_15_H_26_O_2_	238.37
Yield of essential oils (%)	0,15	0,12	0,6	0,22		

**Table 2:** Essential oils monoterpenes compounds of *Artemisia herba alba* (%).

	Leaves	Stems	Capitulum	Aerial parts
Oxygenated sesquiterpenoids	39,89	10,57	22,86	46,15
Sesquiterpenes	29,04		12.92	
Esters	23,97	62,8		
Monoterpenes with pinane skelton	4,48		9,24	26,02
Monoterpenes with thujane skeleton	1,24		10,91	10.78
Monocyclic monoterpenes			21,48	6.59
Monoterpenes with bornane skelton			3,31	8.98
Fatty acid derivate		17.22		

The essential oil extracted from aerial parts (mixture of capitulum and leaves) is very rich in oxygenated sesquiterpenes (46.15%) and pinane skeleton (26.02%), other constituents are representative by monoterpene skeleton with thujane (10.78%), monoterpenes with bornane skeleton (8.98%) and monocyclic monoterpenes (6.59%). In Tunisian *Artemisia herba alba* essential oil, oxygenated monoterpenes and oxygenated sesquiterpenes represent the major fraction of the oil [18].

Thujone was reported as the major component in the essential oil from Tunisia (64%), whereas it was absent in Spain for all the essential oils studied [8, 11]. In this study, thujone concentration was 1.04%. In previous studies, Benjilali and Richard identified seven chemotypes with high concentration varying between 34% and 94% of α and β thujone in *Artemisia herba alba* essential oils collected from different Moroccan regions [19, 21]. Also, in Algeria, α thujone and β thujone were characterized as the major components in the studied oil [22,23]. In our investigation, β thujone concentration was 6.14% but α thujone was not detected in aerial parts oil. On the basis of the results obtained by Ouyahya et al [10] for three Moroccan artemisia oils (*Artemisia negri*, *Artemisia mesatlantica* and *Artemisia herba alba*) it appears that the percentage composition of α and β thujone vary according to the geographical growth site, season, environmental and climatic conditions.

It has been reported in almost all samples described in the literature that cineole is one of the most common constituents in *Artemisia herba alba* essential oil. It has been found as a major component in the essential oil from Spain (Eastern and central Spain) [8, 11], Israel [8], Egypt [24], and in other Moroccan locations [13]. Its concentration reaches high values (superior to 40%) in Spain and Israel [11, 8]. In Tunisia none or very low values were found for 1,8-cineole concentration [15,16], like essential oils from Algeria and Jordan [12, 14]. In this study 1,8-cineole concentration varied between 1.49% in aerial parts and 20.37% in leaves.

Camphor was found at high concentrations in essential oil samples from Southern Tunisia [18] and Algeria (19.14%) [14]. In Morocco, a previous study showed that camphor is one of the most encountered components in *Artemisia herba alba* essential oil. Five chemotypes were defined as camphor type oils [20]. In our work, we found 5.12% of Camphor.

Chrysanthenone is a common compound in oils from Israel, Spain (28.2% -36.4%), Tunisia (17%) and Algeria (22.5% sample from Sidi Aissa) [2], and even from other Moroccan samples (77%). Here it was not detected in aerial parts from *Artemisia herba alba*; it was rather present as a minor concentration in capitulum (1.2%). Among the compounds identified in other *Artemisia herba alba* oils, and not detected in this study, the absence of davanon is worth noting; it has been found to be the major constituent in some populations of *Artemisia herba alba* from other regions of Morocco [21, 25] and as a minor constituent in samples from Israeli [8, 26] and the Sinai desert and surroundings [8, 27]. In Spain, its concentration reaches high values (superior to 39,1%) but it is absent in other essential oils samples [11].

**Fig 2:**
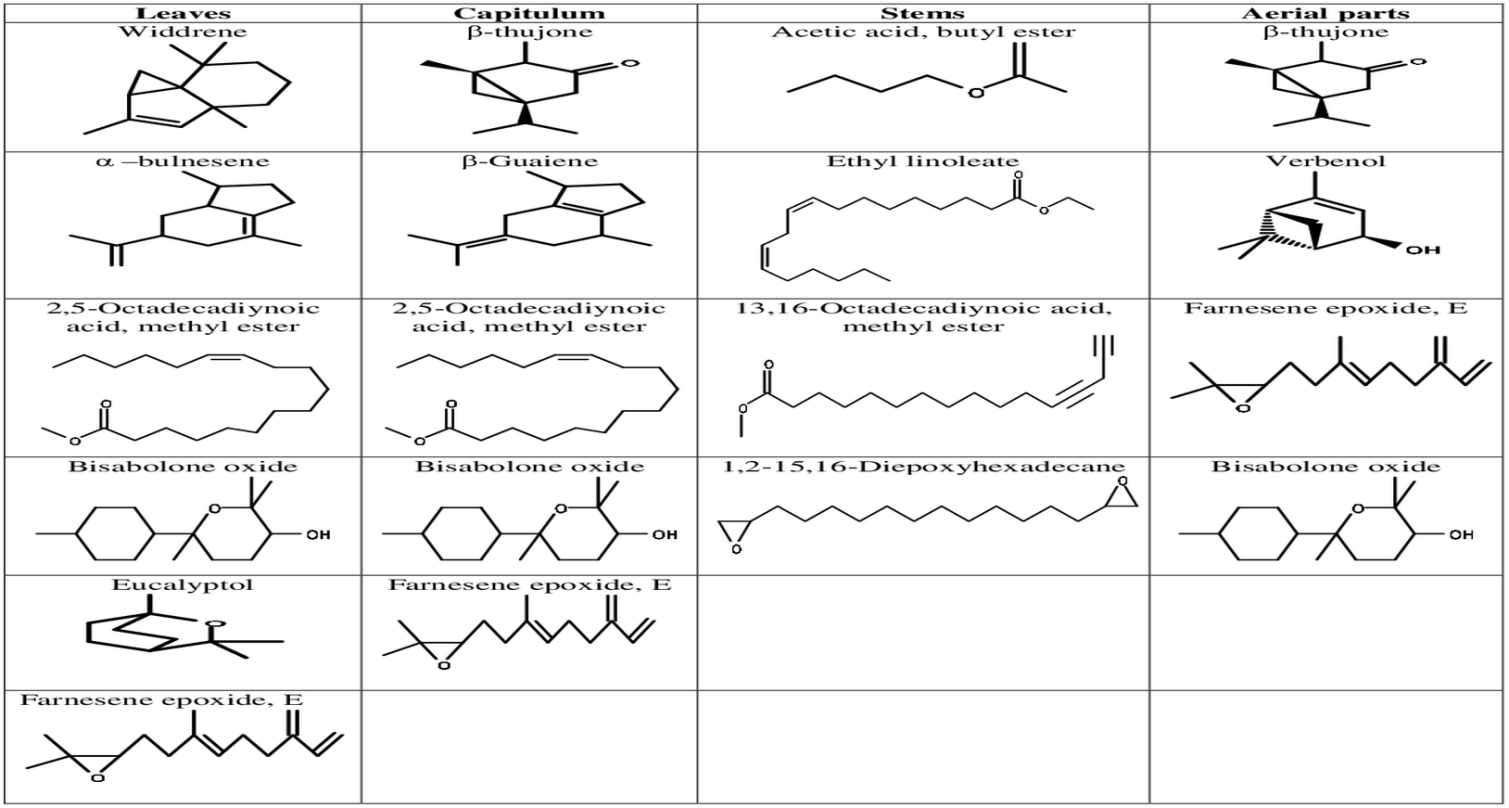
Structures of the most abundant compounds identified in different parts of *Artemisia herba alba* essential oils.

Our results show that essential oils from different botanical parts of *Artemisia herba alba* studied don’t contain camphor, thujone, and 1.8 cineole as the main components like other Moroccan [22], Algerian [2] and Israeli types of essential oils. Furthermore, our study identifies new molecules that have not been cited in other studies and the possible characterization of a new chemothypes (verbenol, trans α bisabolone, Farnesene epoxyde) of *Artemisia herba alba* essential oils (Fig 2). Trans α bisabolone and farnesene epoxyde, have been reported to exhibit potential antitumor activities [28, 29].

Through the study of the essential oils by GC-MS, we have been able to identify and quantify the composition of the essential oils of *Artemisia herba alba*, which varies in different parts of plants. These qualitative and quantitative differences in the chemical composition of essential oils might be attributed to several factors such as geographical factors (location), climatic effects of the plants, harvest season, nature of the soil, age of the plant parts (young or adult), the state of plant material used (dried or fresh), the part of the plant used, time of collection, the extraction process, genetic variability (chemotype), [30] etc.

Our results are in concordance with other reports that demonstrated how plant growth are affected by genetic and environmental factors, and how these factors contribute to differences in the chemical variation of essential oils of plants with different chemotypes [31, 32]. The chemical variation of essential oils of different chemotypes of *Thymus* species from different locations or growing in the same habitat have been attributed to the difference in environmental and genetic factors [31, 33, 34, 35]. Furthermore, ecological factors, climatic characteristics, particularly, light and temperature, have also been reported to influences the production of essential oils as well as other active agents in plants [32, 36, 37, 38, 39].

### Anticancer activity of *Artemisia herba alba* essential oils

Chemotherapy is not giving significant benefits and it is often associated to systemic toxicity and drug resistance. Thus, searching for new therapeutic agents that are able to circumvent the chemotherapy side effects, remains a more efficient to reduce toxicity and increase their effectiveness against cancer. Therefore, we carried out the *in vitro* anticancer activity of essential oils obtained from different biological parts of *Artemisia herba alba* growth in Morocco. *Artemisia herba alba* is a frequent component of herbal mixtures used in the traditional medicine [40, 3, 4].

**Fig 3:**
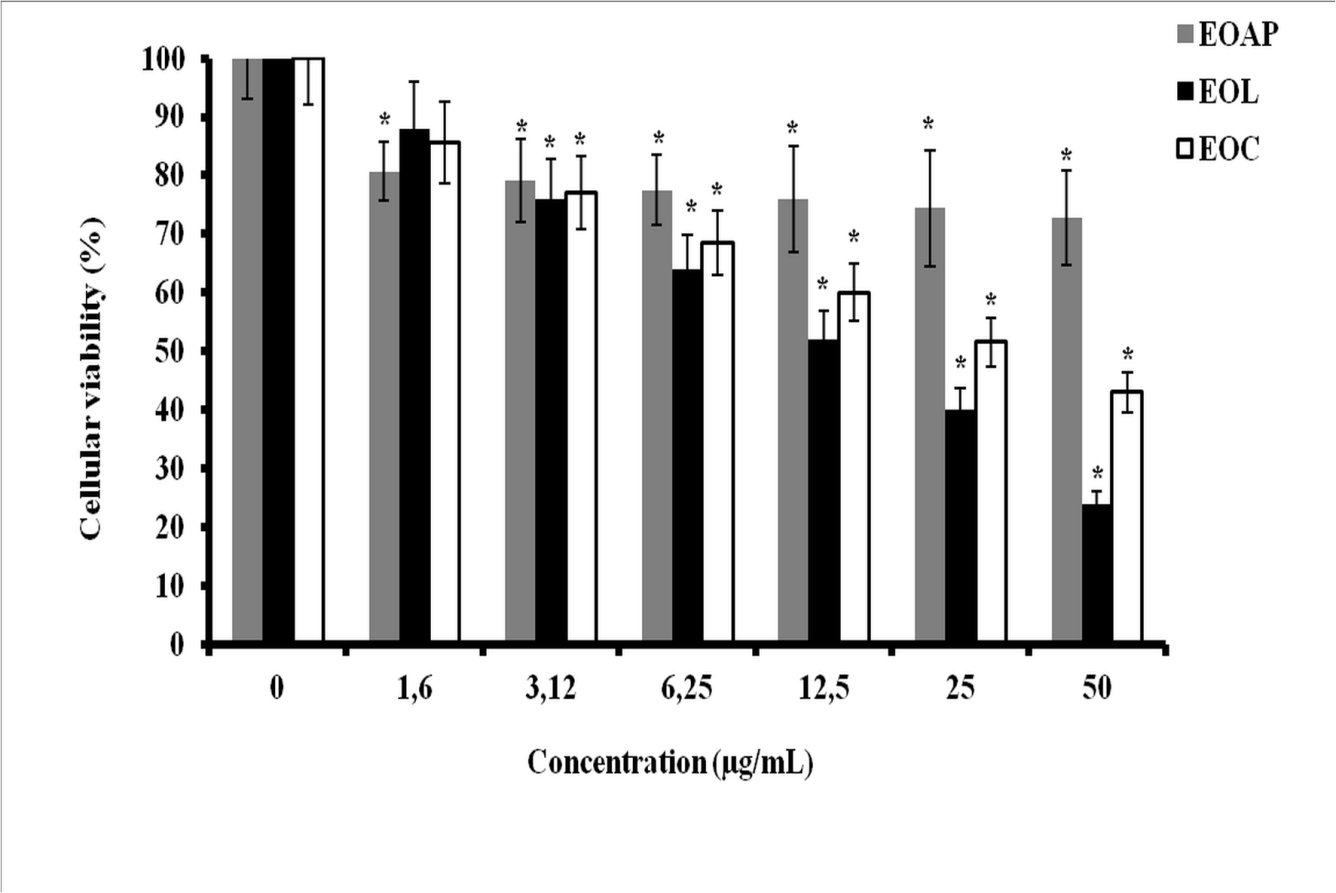
Cytotoxic effect of *Artemisia herba alba* essential oils on P815 cell line. P815 cells treated with different concentrations of essential oils from aerial parts (EOAP), leaves (EOL) and capitulum (EOC). Bars show the mean percentage ± SD. *P<0.05.

**Fig 4:**
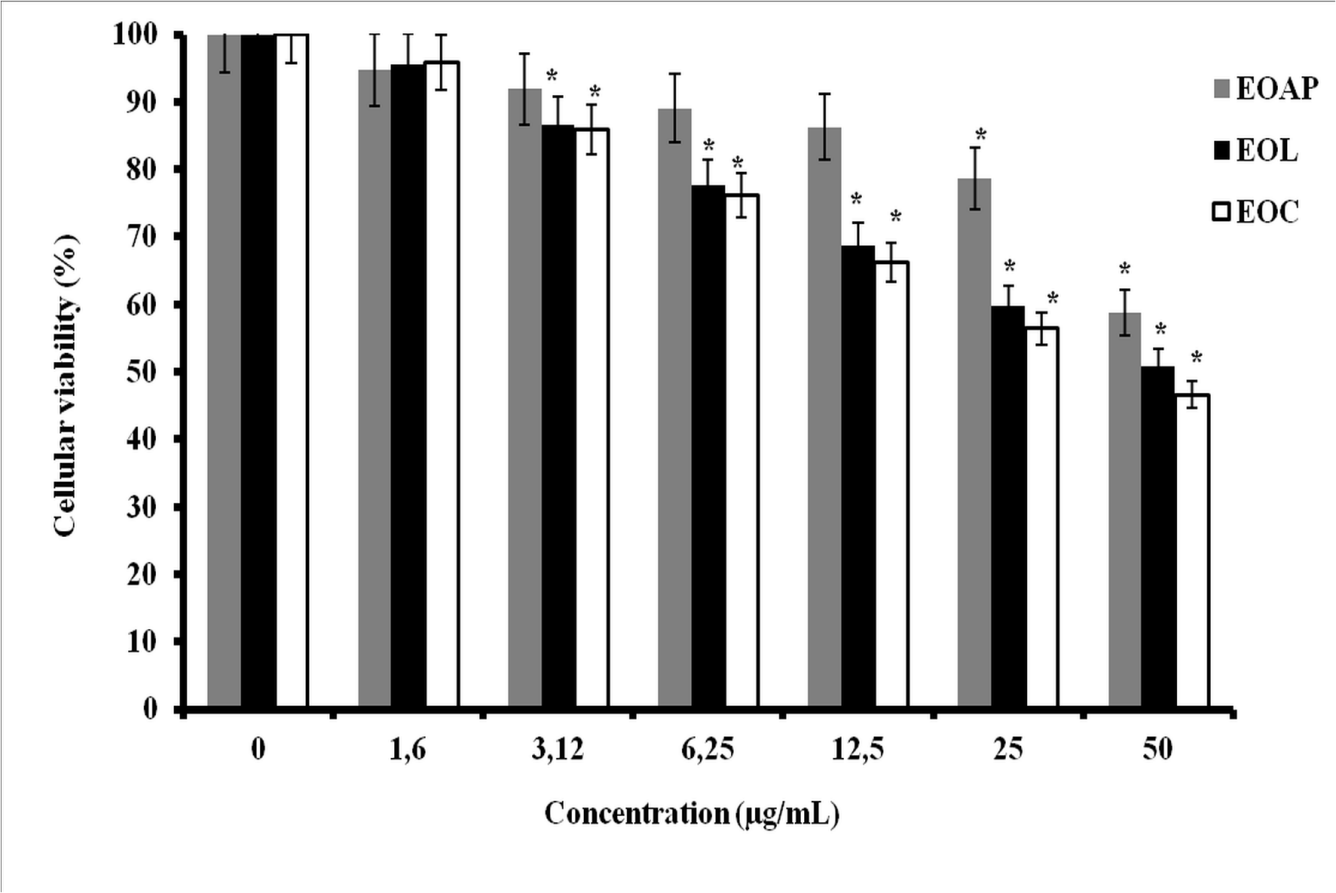
Cytotoxic effect of *Artemisia herba alba* essential oils on BSR cell line. BSR cells treated with different concentrations of essential oils from aerial parts (EOAP), leaves (EOL) and capitulum (EOC) of *Artemisia herba alba* for 48h. Bar graph shows the mean percentage ± SD. *P < 0.05.

*In vitro* anti-tumor cytotoxicity assays of *Artemisia herba alba* oils were performed against P815 and BSR cell lines. These oils include: the essential oils of leaves, capitulum and aerial parts. The results obtained are reported in Figs 3 and 4; it is shown that all essential oils have an important dose dependant cytotoxic effect against P815 and BSR cell lines. However, in P815 cell line (Fig 3) the cytotoxicity decreased in the following order: essential oil of leaves > essential oil of capitulum > essential oil of aerial parts. The concentration leading to 50% cytotoxicity (IC_50_) was about 15μg/ mL (P<0.05) for leaves oil and 36μg/ml for capitulum essential oil, however; the essential oil of aerial parts did not show an important cytotoxicity and did not reach IC_50_ (P<0.05). In BSR cell line (Fig 4), the cytotoxicity induced by essential oils studied shows that capitulum oil (IC_50_ = 20μg/mL, P<0.05)) is slightly sensitive to leaves oil (IC_50_ = 26μg/mL, P<0.05) and more sensitive than aerial parts oils (IC_50_ = 50μg/mL) (P<0.05). The results suggest that the cytotoxicity effect can be attributed to the nature of chemical compound content in essential oils extracted from different botanical parts of *Artemisia herba alba* and on cancer cell lines target.

On basis of literature data it is possible to hypothesize that the cytotoxic effects exhibited by essential oils, under these experimental conditions, could be related to an overall action of the compounds present in oils, especially to sesquiterpene compounds [41, 42]. The essential oil of leaves, which presents a higher concentration of sesquiterpenes (68.93%) and particularly oxygenated sesquiterpenes (39.89%), has been shown to be more active than other essential oils extracted from *Artemisia herba alba*. In fact, the anticancer activities of sesquiterpenes have been reported in the literature. It has been found that α-humulene is active against A-549, DLD-1 and LNCaP cell lines [43, 44, 45]. Also, caryophyllene exhibited antiproliferative activity against K562 cell [46]. Therefore, our results on tumor cell lines suggest that the anticancer activity of the essential oils from different biological parts of *Artemisia herba alba* may be related to active sesquiterpenes associated to the synergism of other natural products present in the essential oil composition.

**Fig 5:**
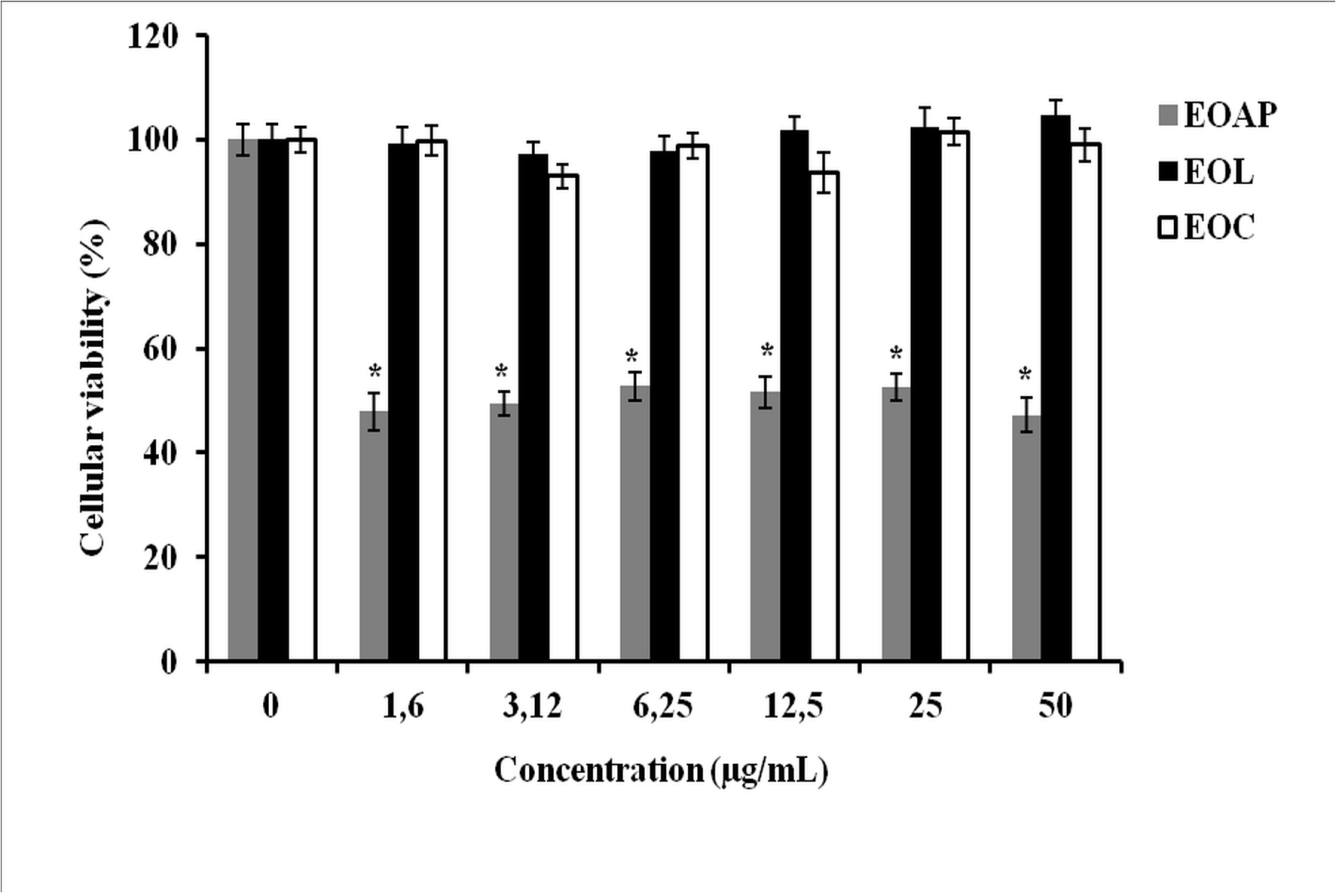
Effect of *Artemisia herba alba* essential oils against the PBMC. PBMC were prepared from human normal donors by Ficoll-hypaque density centrifugation. Cells were incubated in 96-well microtiter plates in the presence of different essential oils (essential oil of leaves: EOL, essential oils of capitulum: EOC and essential oils of aerial parts: EOAP) at different concentrations (0, 4 μg/ml–50μg/ml). After 48 h incubation, viability was determined using MTT assay as described in materials and methods. Bar graph shows the mean percentage ± SD. *P < 0.05.

The difference in cytotoxic activity of essential oils extracted from different biological parts, indicate that cancer cell lines studied differ with respect to their sensitivity to the substances contained in the essential oils of *Artemisia herba alba* and the molecular characteristics of the cells as well. A study of cytotoxicity induced by different essential oils in *Saccharomyces cerevisiae* shows that these cells are more sensitive to *Artemisia herba Alba*, this cytotoxicity is accompanied by the induction of cytoplasmic mutation, indicating mitochondrial damage and impairment of oxidative metabolism [47]. In our study, although the essential oils of some parts of the plant not shown a cytotoxic effect against the PBMCs (EOC, and EOL), some of them demonstrate a cytotoxic effect on PBMCs (EOAP). On the other hand, the microscopic comparison of the viable cancer cells and the PBMCs treated with 50μg/mL by the essential oils from leaves and capitulum (EOC and EOL), shown after 24h lysis of tumor cell lines (70%) using the bleu trypan exclusion method. Whereas, the PBMCs don’t demonstrate the bleu coloration reflecting their viability, this observation was not shown after treatment with the EOAP which demonstrate a lysis effect toward the PBMCs (Fig 5). Moreover, Tilaoui et al, (2011) reported the cytotoxic activity of aerial part essential oil of *Artemisia herba alba* on CEM cancer cell lines with IC_50_ = 6μg/mL [40]. The cytotoxicity of *Artemisia herba alba* essential oils could be related to the production of reactive oxygen species (ROS). It is reported that exposure to essential oils strongly affects the cell wall and membranes and damages mitochondria. This may lead to mitochondrial dysfunction and to a radical burst of reactive oxygen species that triggers gene induction and apoptotic cell death [48, 49]. In fact, mitochondrial dysfunction is known to increase intracellular concentrations of DNA-damaging species such as superoxide and peroxide ions linked to apoptotic death [50, 51].

Earlier studies have shown that plants are known to display variation in the concentration of the bioactive phytochemicals depending on intrinsic factors like the age of the plant, and on extrinsic factors like the geographical climate, circadian rhythm, the nature of the soil, and the season. The cytotoxic activity of essential oils might be due to the synergic effects of all the terpenes in the essential oil, or perhaps there are some other active compounds responsible for the antiproliferative activity of the essential oil, which deserves attention in any future study [52, 53]. However, it must be kept in mind that essential oils are complex mixtures of numerous molecules, and one might wonder if their biological effects are the result of synergism among all molecules or reflect only the activities of the main molecules present at the highest concentrations according to the gas chromatographic analysis. Generally, the major components are found to reflect quite well the biophysical and biological features of the essential oils from which they were isolated, with the amplitudes of their effects being dependent on their concentration when they are tested on their own or in essential oils [54]. Also, the differential effects observed between cancer cell lines could be related on the target of these cells, as P815 is a suspension cell line and BSR is an adherent cell lines. Our result is in accordance with Kumura et al, (2004), who demonstrate that inhibition of cell proliferation by Methotrexate is different in suspension (FM3A, 2B4 and THP-1) and adherent (NIH3T3 and V79) cells [55].

### Effect of *Artemisia herba alba* essential oils against PBMCs

**Fig 6:**
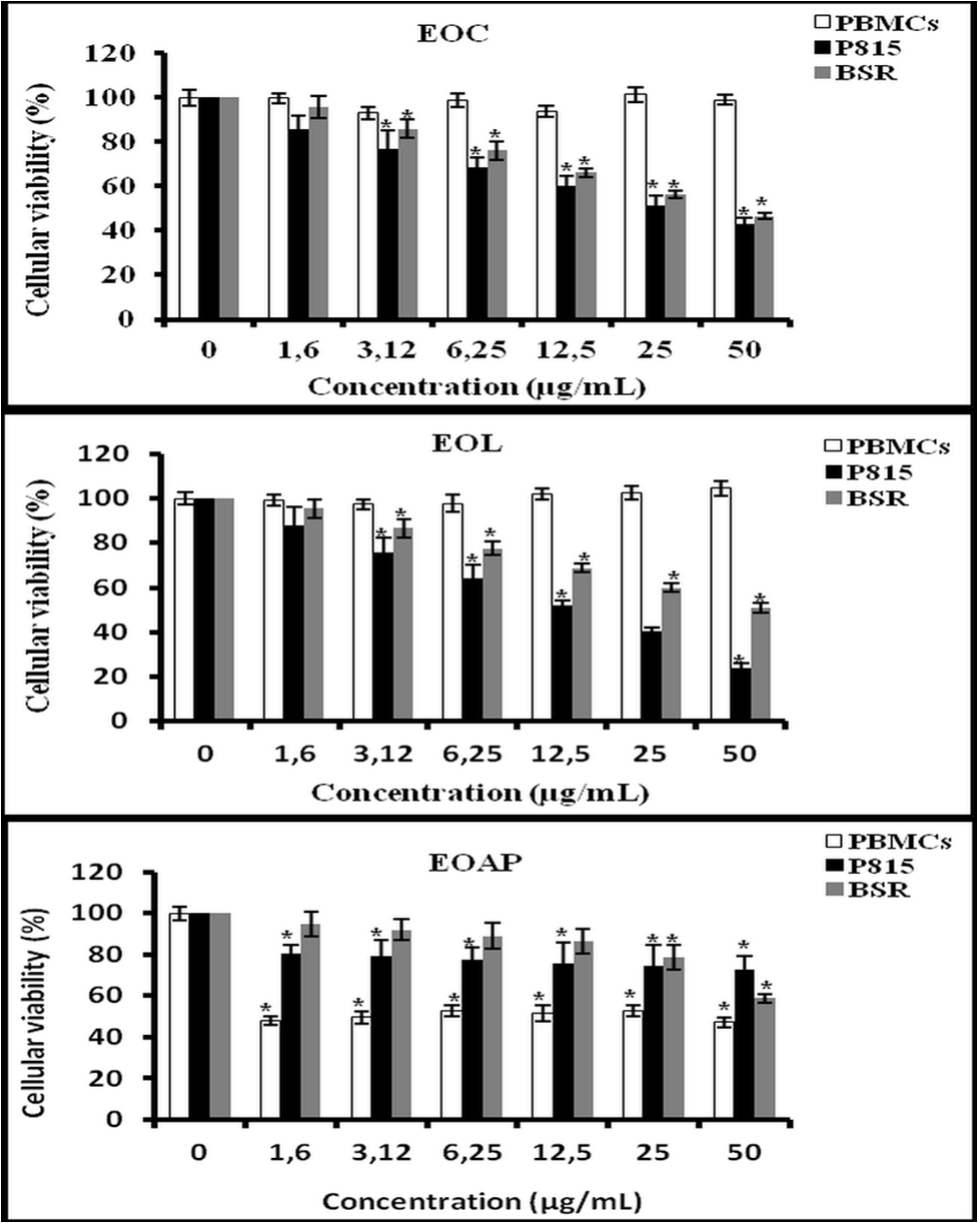
Effect of essential oils from different organs plant of *Artemisia herba alba* against normal cells (PBMCS) and cancer cell lines (P815, BSR). EOC: essential oils of capitulum; EOL: essential oils of leaves; EOAP: essential oils of aerial parts (mixture of leaves and capitulum). Bar graph shows the mean percentage ± SD. *P < 0.05.

The majority of clinically approved anticancer drugs are characterized by a narrow therapeutic window that results mainly from a high systemic toxicity of the drugs in combination with an evident lack of tumor selectivity [56]. In this connection, we tested our essential oils against the human peripheral blood mononuclear cells (PBMC) in order to determine their effects against normal cells. The results obtained are represented in Fig 5. It is depicted in this figure that the essential oil of aerial parts causes around 50% of cytotoxicity into PBMC. However, the essential oils of leaves and capitulum were shown at a concentration as being able to induce a cytotoxic activity against tumor cells (P815 and BSR); no cytotoxicity effect was observed (Fig 6) but, instead of that, leaves and capitulum oils produced a slight proliferative effect on normal PBMC with 105% and 102% of viability (P<0.05) after 48 h of treatment for leaves and capitulum respectively. (Fig 5). The differential cytotoxic effect toward PBMCs and cancer cells (Fig 6) could be explained by the difference of the metabolism of each cell line type. Also, it might be due to the differential expression of the genes and consequently the modulation of the activity of essential oils. Similar observations *in vivo* are also known, where it has been seen that a plant extract exhibits antiperoxidative and pro-oxidative effect for cardiac and liver tissues respectively [57].

## Conclusion

In Summary, the present work confirms the very important chemical variability in *Artemisia herba alba* essential oils. As far as we could investigate, such study can be considered as the deep detailed report on the chemical composition and the *in vitro* anticancer activity of the essential oils extracted from stems, leaves, capitulum and mixture of capitulum and leaves of *Artemisia herba alba*. Furthermore, this study identifies new molecules that have not been cited in other studies and the possible characterization of new chemotypes of *Artemisia herba alba* essential oils. The antitumor activity of the essential oils from different biological parts of *Artemisia herba alba* has been determined comprehensively against two cancer cells lines P815 and BSR. Overall, P815 cell lines are more sensitive than BSR cell lines to essential oils effect; our results suggest that leaves and capitulum essential oils are more cytotoxic than aerial parts studied. However, no cytotoxic effect of these essential oils was observed on the human normal cells, apart from a slight proliferative effect. It seems likely that *Artemisia herba alba* could play an important role in combating cancer. Clearly, determining the bioactive compound(s) in *Artemisia herba alba* essential oils that inhibit cancer cell lines proliferation will need further investigation, to clarify the mechanism of action and their potential use.
